# Genetic regulation of exosome biogenesis pathway in human adipose and muscle tissue and association with obesity and insulin resistance

**DOI:** 10.21203/rs.3.rs-6580546/v1

**Published:** 2025-07-11

**Authors:** Swapan Das, Gagan Deep, Mary Comeau, Carl Langefeld

**Affiliations:** Wake Forest University School of Medicine; Wake Forest University School of Medicine; Wake Forest University School of Medicine; Wake Forest University School of Medicine

## Abstract

**BACKGROUND:**

Animal studies provide evidence of a link between exosome profile, obesity and insulin resistance (IR). Although it is known that exosomes mediate cell-cell communication via their macromolecular cargo, the factors regulating exosomes in humans are unknown.

**METHODS:**

Leveraging genome-wide expression and genotype data from the African American Genetics of expression and Metabolism (AAGMEx) cohort, we focused on 262 genes in “Exosome pathway”, curated by us, to examine the relationship of the expression of these genes with IR and obesity and tested the role of single nucleotide polymorphisms (SNPs) in determining the variability in the expression of these genes in adipose and muscle tissue. Publicly available gene expression data on European ancestry individuals, genome-wide association studies (GWAS), and bioinformatic approaches were used to validate the role of obesity-associated genetic variants in regulating exosome pathway genes.

**RESULTS:**

Transcript levels of 96 and 15 exosome pathway genes were associated with gluco-metabolic traits (BMI and insulin sensitivity) in adipose and muscle tissue, respectively. Data also suggests transancestral replication of association. The *cis*-expression quantitative trait (cis-eQTL) analysis of exosome pathway genes identified 45 and 65 *cis*-eGenes in adipose and muscle tissue, respectively. Expression of a subset of 26 *cis*-eGenes in adipose were also associated with gluco-metabolic traits. Based on combined SNP-to-gene-linking analysis 35 and 82 adipose expressed exosomal genes (e.g. *AHNAK, RAP2A*) were identified as target genes for gluco-metabolic trait-associated SNPs in GWAS catalogue and UKBB GWAS datasets, respectively.

**CONCLUSIONS:**

In summary, expression of exosome pathway genes in adipose and muscle tissue are associated with obesity and IR, and expression of a subset of these genes are determined by SNPs. Furthermore, analysis of the target genes of GWAS identified gluco-metabolic trait-associated SNPs suggests that a subset of these SNPs is potentially involved in causing obesity and related gluco-metabolic diseases, likely by modulating exosome biogenesis.

## Introduction

Systemic dysregulation of tissue cross-talk and cell-cell communication play important roles in the pathogenesis of many metabolic diseases, including obesity and type 2 diabetes^1, 2^. Cells secrete bioactive components, and these components play an important role in endocrine as well as paracrine communication between cells^3–6^. Cellular secretome is a commixture of soluble factors as well as molecules associated with extracellular vesicles of various sizes and complexities. Small extracellular vesicles (sEV) are lipid bilayer delimited particles of < 200nm diameter released from cells^7^, present in all biofluids^8^ and transport various molecules, including proteins, RNAs and metabolites related to the cell of origin and their physiological/metabolic state^9–12^. sEV contents are protected while in circulation, making them an efficient means for intercellular communication. Evidence from recent studies suggests the ability of metabolically active tissues, including adipose and muscle, to release sEV, and these sEV can act locally or enter the circulation to have systemic effects^13–19^. Studies on tissue-derived sEV in human are limited, but recent studies by us^20^ and others^21–23^ confirmed the presence of adipose- and muscle tissue-derived sEV in human plasma, and indicted their role in tissue-crosstalk.

Biogenesis of a major subset of these sEV takes place in the internal compartments of the cell (as intraluminal vesicles, ILVs), and they are released via the fusion of multivesicular bodies (MVBs) with the plasma membrane and are termed “exosomes”^24–27^. Tetraspanin positive, specially CD63-postive exosomes constitutes the major fraction of secreted exosomes^24^. Exosome biogenesis is a complex, highly regulated process that involves several sequential stages, from the initial formation of ILVs within the endocytic compartments of MVBs to the eventual release of fully mature exosomes in the extracellular environment^28, 29^. The ILVs interact with trafficking effectors, which lead to membrane bending and scission processes, giving rise to mature exosomes. The commonest and first identified mechanism that regulates ILV formation is known to be regulated by the endosomal sorting complexes required for transport (ESCRT) machinery and accessory protein the ATPase VPS4. Alternative ESCRT independent mechanism for ILV formation includes syndecan – syntenin - ALX mediated mechanisms and processes involving ceramides generated from sphingomyelin by neutral sphingomyelinase (nSMase)^28–31^. The exosome biogenesis process is intertwined with the selective sorting and packaging of cargos within exosomes and dictates the target specificity and functional roles of exosomes. Thus, understanding the regulatory mechanisms determining exosome formation is crucial for understanding their role in disease biology^28^. Recent studies in animal and cell culture models, and studies examining rare, inherited coding variants in humans suggest a role for genetic factors in determining the biogenesis of sEV, including exosomes^32–35^. However, genetic factors regulating biogenesis of sEV and their relationship in metabolic diseases in humans remain obscure.

We hypothesize that the expression of genes involved in the molecular machinery of biogenesis, assembly, and secretion of exosomes in metabolically active tissues are associated with insulin resistance, obesity, and related diseases in humans, and the variability in the expression of these genes are partly determined by genetic regulatory variants. In this study, we tested these hypotheses by leveraging the genotype and adipose and muscle tissue transcript expression data from the phenotypically well-characterized African American Genetics of Metabolism and Expression cohort (AAGMEx)^36, 37^. Results from our analysis in AAGMEx cohort, corroborated in other cohorts support the role of genetic factors in exosome biogenesis.

## Methods

### Human subjects and physiological phenotyping

Multiomics data including, adipose and muscle tissue gene expression and genotype data from participants in the African American Genetics of Metabolism and Expression (AAGMEx) cohort, recruited previously by us, was used in this study. The AAGMEx is a cohort of 256 metabolically well-characterized individuals without diabetes from North Carolina. Clinical, anthropometric, and physiological characteristics of the AAGMEx cohort have been previously described^36, 37^. Briefly, cohort participants were healthy, self-reported African American men and women (53.9% male) residing in North Carolina, aged 18 to 60 years (mean age =40.7 ± 11.5 years), with a BMI between 18 and 42 kg/m^2^ (mean BMI =28.9 ± 5.6 kg/m^2^). During the first clinic visit, fasting blood samples were drawn for biochemical analyses and DNA isolation. A standard 75-g oral glucose tolerance test (OGTT) was used to evaluate insulin sensitivity and exclude individuals with diabetes. Plasma glucose levels in the AAGMEx cohort were analyzed by glucose oxidase methods at a CLIA-certified commercial laboratory (LabCorp, NC,USA). Plasma insulin was measured using an immuno-chemiluminometric assay (Invitron Limited, Monmouth, UK). Plasma glucose and insulin data from five OGTT time points (0, 30, 60, 90, and 120 min) were used to calculate the Matsuda insulin sensitivity index (http://mmatsuda.diabetes-smc.jp/MIndex.html; in AAGMEx, mean Matsuda index =6.2 ± 6.7). Non-diabetic individuals were invited for the second visit, and abdominal subcutaneous adipose near the umbilicus and *vastus lateralis* skeletal muscle biopsies were obtained under local anesthesia. Tissue biopsies were collected by Bergstrom needle from participants after an overnight fast. Tissues were immediately rinsed in sterile saline, quick-frozen in liquid nitrogen, and stored at −80°C. Detailed evaluation of insulin sensitivity was performed during the second visit by the insulin-modified (0.03 U/kg) frequently sampled intravenous glucose tolerance tests (FSIGT). The MINMOD Millennium program^38^ was used to analyze FSIGT data to determine insulin sensitivity index (S_I_) by Minimal model analysis (in AAGMEx, mean S_I_ =4.0 ± 3.3). All participants provided written informed consent under protocols approved by the Institutional Review Board at Wake Forest School of Medicine. In addition, we used publicly available data from cohorts of European ancestry for validation of our findings (see Statistical and bioinformatic analysis sections below).

### Curation of human exosome pathway

Gene ontology and pathway databases curate gene lists in biological pathways based on experimental, genetic, computational, and other validation data. However, an annotated list of exosome biogenesis pathway genes was not available for humans from public databases. Thus utilizing a mouse gene ontology browser (https://www.informatics.jax.org/vocab/gene_ontology), we first curated a list of 197 mouse genes which includes genes involved in biogenesis, assembly and secretion in the cellular component exosome (GO:0070062: extracellular exosome). We further utilized HGNC Comparison of Orthology Predictions (HCOP) search tool (https://www.genenames.org/tools/hcop/), developed by HUGO Gene Nomenclature Committee (HGNC), to convert the mouse gene list to an annotated gene list for human. Our final annotated human “exosome pathway” gene list included 262 unique genes with entrez gene ID **(Supplementary Table-1)**.

### Adipose and muscle transcript expression profiling

Extraction of total RNA from adipose and muscle was performed using miRNeasy Mini Kit (Qiagen, USA) and Ultraspec RNA total RNA extraction reagent (Biotecx Laboratories Inc., Houston, TX), respectively. Quantities of RNA samples were determined by ultraviolet spectrophotometry (Nanodrop, Thermo Scientific), and the quality of RNA was determined by electrophoresis (Experion nucleic acid analyzer, BioRad Laboratories, Inc.). Genome-wide expression data were generated using HumanHT-12 v4 Expression BeadChip (Illumina, San Diego, CA). Participants were block-randomized by age, gender, and BMI, and their RNA samples were assigned to a group, totaling 12 samples for hybridization per BeadChip. Chips were scanned in the Illumina HiScan Reader using Illumina iScan Control Software. Genome-wide gene expression data (probe level) were extracted using Illumina GenomeStudio V2011.1. Expression levels were log_2_ transformed, robust multi-array average normalized (RMA, includes quantile normalization), and batch-corrected using ComBat. The HumanHT-12 v4 Expression BeadChip includes 47,231 probes annotated to transcripts; however, data on transcript probes encompassing common SNPs (based on ReAnnotator, or SnpInProbe annotation, and UCSC SNPv141) and transcripts that were not significantly expressed (p-value<0.05) in ≥25% of the samples were excluded (Data submitted to Gene Expression Omnibus: GEO id #GSE95674 and #GSE95675 in super series #GSE95676)^36, 37^. This transcriptome-wide expression data allowed to test the expression of 246 exosome pathway genes in subcutaneous adipose tissue in the AAGMEx cohort, and we detected 167 genes that were well expressed above background in adipose tissue. Similarly, transcriptome-wide expression analysis of skeletal muscle tissue tested 256 exosome pathway genes of which 167 genes were well expressed.

### Genotyping

DNA was isolated from whole blood using the Gentra Puregene blood kit (Qiagen). DNA samples were measured by NanoDrop and concentrations were adjusted for genome-wide genotyping. As described previously, Infinium HumanOmni5Exome-4 v1.1 DNA Analysis BeadChip (Illumina) and Infinium LCG Quad Assay kits were used to genotype DNA samples (400 ng per subject) based on the manufacturer’s instructions. The Illumina HiScan System was used to scan the BeadChips. Genotype data were examined to verify sample and SNP quality. Samples were excluded if they had a call rate <90% or excess heterozygosity (F <−0.10). Genetic markers were considered high quality if call rates were >95% without departure from expected Hardy-Weinberg proportions (P > 1 × 10^−6^). Identity-by-descent statistics computed by the program KING were examined and did not reveal unexpected duplicates or first- or second-degree relatives. HapMap Phase 3 CEU, YRI, and CHB samples were merged with study samples and admixture estimates were computed using the software ADMIXTURE^39^. Samples with >50% European ancestry proportion were excluded. Genotype assays of 4,210,443 SNPs passed technical quality filters. The genotype of 2,296,925 autosomal SNP assays (representing 2,210,735 unique high-quality genotyped SNPs with MAF > 0.01 and HWE-p value > 1 × 10^−6^) was used in in our published adipose tissue expression quantitative trait (eQTL) analyses^36^. We imputed these genotyped SNPs to the 1000 Genomes dataset (1KGP, phase 3 cosmopolitan reference panel) using the genotype imputation program Minimac3, implemented on the Michigan Imputation Server (https://imputationserver.sph.umich.edu/). The expanded genotype data set includes 14,502,313 autosomal genotyped and imputed SNPs^40^. In this study, we have used this expanded genotype data to determine association between the levels of transcripts expressed in adipose and muscle and the genotype of local SNPs (*cis*-expression quantitative trait loci; *cis*-eQTL) only for selected genes in exosome biogenesis pathway.

### Statistical analyses

We tested for associations between insulin sensitivity and obesity with expression levels of genes in adipose and muscle tissue of the participants in the AAGMEx cohort. To test for associations, we computed linear regression models with the Matsuda index, S_I_ (natural log transformed) or BMI (square root transformed) as the outcome and transcript expression level (log_2_) as the predictor. Models included age, sex, and African ancestry proportion (admixture estimates from genotype) as covariates. We computed p-values adjusted for Benjamini-Hochberg false discovery rate (q-value). Expression of a transcript for exosome pathway genes correlated with Matsuda index or other gluco-metabolic traits at q<0.01 was considered significant. Spearman’s semi-partial correlation analysis was computed between the glucose homeostasis or obesity traits and transcript expression adjusted for age, sex and ancestry proportion (admixture). The square of the partial correlation estimate provides an estimate of the proportion of variation explained by the transcript, accounting for the covariates. The *cis*-expression quantitative trait (*cis*-eQTL) analysis was conducted to identify genetic regulation of “exosome pathway” transcripts in adipose and muscle. For each transcript probe in AAGMEx, we computed linear regression with the log_2_ transformed expression value as the outcome and an additive genetic model for the genotype of single nucleotide polymorphisms (SNPs) as predictors as implemented in the R-package MatrixEQTL, with age, sex, and African ancestry proportion as covariates. This *cis*-eQTL analyses first considered directly genotyped SNPs and later considered high quality imputed SNPs within ±1Mb 5’ and 3’ of respective transcript. *Cis*-eQTLs for exosome pathway genes with a q-value <0.01 were considered significant. To replicate our findings in AAGMEx African Americans, summary statistics of previously completed analyses on adipose^41, 42^ and muscle tissue^43^ data sets for independent cohorts of European ancestry were obtained from publicly available data source.

### Bioinformatic analyses

Genome-wide association studies (GWAS), and meta-analysis of GWAS identified genetic variants associated with obesity and other complex gluco-metabolic traits, however, efforts are needed to improve our understanding on the role of these genetic variants in modulating cellular and molecular mechanisms involved in obesity^44^. We hypothesize that a subset of SNPs identified in obesity and gluco-metabolic trait GWAS may be mechanistically involved in modulating the expression of genes in “exosome pathway’. To determine if the adipose expressed “exosome pathway” genes are also target genes for obesity and related gluco-cardio-metabolic trait-associated SNPs found in GWAS datasets, we interrogated the list of predicted target genes for these trait-associated SNPs determined by Gazal S et al. (2022) utilizing SNP-to-gene-linking strategies^45^. In this strategy trait-associated SNPs were connected with their target genes based on several layers of functional genomic information and various SNP-to-gene-linking (S2G) methods and provided a combined S2G (cS2G) score for each gene. Genes with cS2G score >0.5 (data source: https://alkesgroup.broadinstitute.org/cS2G) were selected as significant target genes, as recommended. As a complementary method we also used the list of target genes for GWAS identified obesity and related gluco-cardio-metabolic trait-associated SNPs prioritized by polygenic priority score (PoPS) method (data source; https://www.finucanelab.org/data)^46^.

## Results

### Expression of exosome pathway genes in adipose and muscle tissue are associated with obesity and insulin sensitivity:

**Figure 1:**
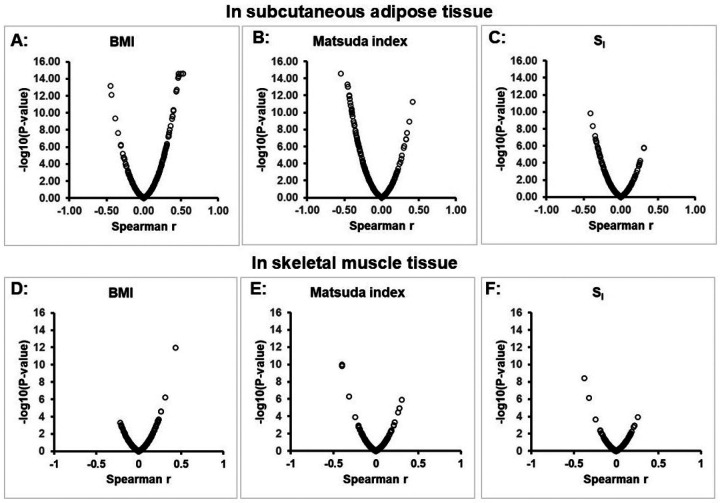
Expression of “exosome pathway” genes are correlated with obesity and gluco-metabolic traits. Scatter plot showing the correlation of BMI, Matsuda index (insulin sensitivity index from OGTT) and S_I_ (insulin sensitivity index form FSIVGT) with the expression (transcript levels) of exosome pathway genes in subcutaneous adipose (A, B and C) and skeletal muscle tissue (D, E and F) of African Americans in AAGMEx cohort. Each dot represents a transcript probe for an exosome pathway gene. The −log10 (p-value) and r is the uncorrected statistical significance and correlation from Sperman’s semi-partial correlation analysis, adjusted for age, sex, and ancestry proportion (admixture). P-value<2.66 ×10^−15^ were truncated for presentation.

Among the genes in our curated human “exosome pathway” (*i.e*., genes putatively involved in biogenesis, assembly and secretion of exosomes), we identified reliable expression of transcripts from 167 unique genes (with Entrez id) in adipose tissue of AAGMEx cohort participants. The transcript level of 96 of these 167 genes were significantly associated (q-value <0.05 in linear regression analysis; **Supplementary Table-2)** with obesity (BMI) and/or gluco-metabolic traits (Matsuda Index and S_I_). As shown in Figure-1A–C, complementary non-parametric Spearman’s semi-partial correlation analysis also supported the correlation of the expression of obesity and gluco-metabolic traits with expression of exosome pathway genes in adipose tissue. Among the BMI-associated exosome pathway genes, 53 was positively correlated while 28 was negatively (inversely) correlated. Top genes positively correlated with BMI includes *RAP2A* (b= 1.57, P= 9.81 ×10^−21^, Spearman’s partial correlation r= 0.526) and *CD86* (b= 1.15, P= 5.02 ×10^−18^, r= 0.531), while the top genes inversely correlated with BMI include *ACY1* (b= −1.1, P= 4.2 ×10^−15^, r= −0.449) and *HSPD1* (b= −1.18, P= 8.8 ×10^−13^, r= −0.432); thus these transcripts explained approximately 20% of the residual variation in BMI after accounting for the covariates. Using regression analyses results based on adipose tissue transcript expression data from METSIM, a European ancestry cohort of 770 male individuals from Finland^42^, we sought replication of BMI and Matsuda index–correlated transcripts identified in AAGMEx African Africans. Expression of a total of 105 exosome pathway genes in adipose tissue of METSIM cohort participants were significantly associated (P_FDR_ < 0.05) with BMI and/or MATSUDA insulin sensitivity index **(Supplementary Table-3)**. The BMI was positively and inversely correlated with the expression of 44 and 19 exosome pathway transcripts in both cohorts, respectively. For example, *RAP2A* was strongly positively associated (b = 0.37, P_FDR_ = 5.35 × 10^−23^) and *HSPD1* was inversely associated (b = −0.32, P_FDR_ = 1.72 × 10^−17^) with BMI in METSIM. Thus, our results suggest transancestral replication of association and direction of effect for 77.7% of BMI-correlated exosome pathway transcripts in independent cohorts of African and European ancestry individuals.

In contrast to subcutaneous adipose tissue, transcript expression levels of only 15 exosome pathway genes in skeletal muscle tissue of AAGMEx participants were significantly associated (q-value <0.05) with gluco-metabolic traits (BMI, Matsuda Index and/or S_I_), 8 positively correlated and 4 negatively correlated with BMI **(Supplementary Table-4)**. As shown in Figure-1D–F, Spearman’s semi-partial correlation analysis also supported these findings. Top genes positively correlated with BMI include COPS5 (b= 1.28, P= 7.83 ×10^−14^, r= 0.432) and *MB* (b= 0.67, P= 1.57 ×10^−6^, r= 0.31), with the top gene *CHMP2A* negatively correlated with BMI (b= −0.819, P= 4.87 ×10^−5^, r= −0.218). Publicly available linear regression analyses results based on skeletal muscle expression data set from 301 Finnish European individuals in the Finland-United States Investigation of NIDDM Genetics (FUSION) cohort^43^ identified significant association (q-value< 0.05) for the transcript levels of 28 exosome pathway genes with BMI **(Supplementary Table-5)**. Expression of three exosome pathway genes in skeletal muscle, namely *HSPA2, NUCB2*, and *COPS5* was significantly positively corelated with BMI in both AAGMEx and FUSION.

### Expression of exosome pathway genes are associated with the genotype of common genetic variants:

**Figure 2:**
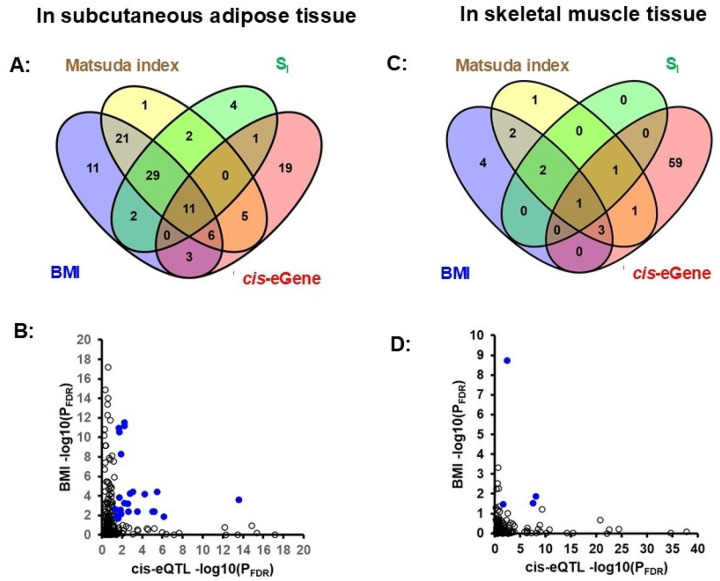
Expression of a subset of obesity and gluco-metabolic traits correlated “exosome-pathway” genes are genetically regulated. Venn-diagrams showing overlap of obesity and gluco-metabolic trait correlated and genetically regulated (*cis*-eGenes) exosome pathway genes in adipose (A) and muscle (C) from AAGMEx cohort. Scatter plot shows the relation between FDR corrected −log 10 (P-value) of gene for association with BMI (from linear regression) and association with genotype (for top SNP from *cis*-eQTL analysis) in adipose (B) and muscle (D). Each dot represents a transcript probe for an autosomal exosome pathway gene for which cis-eQTL was computed. Transcript probes whose expression is significantly associated (P_FDR_<0.05) with BMI and genotype of a local (cis) SNP are shown as blue dots.

The *cis*-expression quantitative trait (*cis*-eQTL) analysis in AAGMEx cohort tested the association of the genotype of local SNPs with the transcript level of 153 adipose tissue-expressed autosomal genes in the exosome pathway. In the directly genotyped SNPs we identified 45 significant cis-eGenes (P_FDR_ <0.05; **Supplementary Table-6)**, suggesting a genetic regulation of the transcript level of these exosome pathway genes. Top *cis*-eGenes in adipose includes *EXOSC1* (top cis-eSNP rs29001317_T, b= −0.299, p= 2.94 ×10^−22^), NSUN2 (rs2303711_T, b= 0.102, p= 2.31 ×10^−20^), and *SDCBP* (rs10102400, Chr8: 59462042_T, b= 0.37, p= 7.29 ×10^−20^). Additional analysis with the genotype of imputed SNPs identified stronger genetic association signal for 29 of these *cis*-eGenes. Publicly available summary statistics of adipose tissue eQTL in 2,344 individuals (includes 2,256 European ancestry individuals from METSIM, FUSION, TwinUK and GTEX cohorts)^41^ validated 42 of the 45 “exosome pathway” *cis*-eGenes identified in AAGMEx African Americans **(Supplementary Table-7)**. This metanalysis data validated cis-eQTL mediated regulation of the expression of several exosome component genes including *EXOSC1, 2, 3, 6* and *7*. As shown in Figure 2A–B, among the exosome pathway cis-eGenes, expression of 26 genes in adipose were also associated significantly with gluco-metabolic traits in AAGMEx cohort, and includes *STEAP3, EXOSC2*, and *EXOSC7*.

The *cis*-eQTL analysis of 153 muscle tissue-expressed autosomal genes in the “exosome pathway” identified 65 significant *cis*-eGenes (P_FDR_ <0.05) in AAGMEx cohort **(Supplementary Table-8)**. Top *cis*-eGenes in muscle includes *DDX11* (top cis eSNP rs7309189_G, b= 0.102, p= 2.03 ×10^−43^), and *EXOSC6* (rs4985407_A, b= 0.196, p= 2.73 ×10^−40^). Among the exosome pathway *cis*-eGenes, expression of only six genes (Figure 2C–D) in muscle were also associated significantly with gluco-metabolic traits in AAGMEx (includes *SERPINA5, HSPA2, COPS5* and *EXOSC9*). The eQTL analyses identified *cis*-regulatory variants for 26 “exosome pathway” genes in both adipose and muscle tissue of AAGMEx African Americans, which suggest that genetic regulation of “exosome pathway” includes shared and tissue-specific components.

### Adipose expressed exosome pathway genes are target genes for obesity and related gluco-cardio-metabolic trait-associated SNPs identified in GWAS datasets:

Utilizing layers of functional genomic information and the combined SNP-to-gene-linking (cS2G) strategy Gazal et. al.^45^ predicted target genes for gluco-metabolic trait-associated SNPs identified in GWAS. We interrogated this list to determine if the “exosome pathway” genes are targets and likely affected by these phenotype or disease-associated genetic polymorphisms. Among the adipose expressed exosome pathway genes, based on cS2G analysis 35 and 82 genes were identified as target genes for gluco-metabolic trat-associated SNPs in GWAS catalogue and UK biobank (UKBB) GWAS dataset, respectively. Transcript level expression of a subset of 53 target gene members of exosome pathway in adipose tissue of AAGMEx cohort were also associated with gluco-metabolic traits **(Supplementary Table-9)**. In Table-1 we show the cS2G predicted target genes of selected obesity (BMI, WHR etc.) associated SNPs curated in GWAS catalogue, and are members of exosome pathway. For example, *AHNAK* was identified as target gene for WHR associated SNP rs2509963 (cS2G score= 1) in GWAS catalogue and WHR associated SNP rs2509970 (cS2G score=1) in UKBB GWAS dataset. In a complementary approach, Weeks et al^46^ also predicted list of target genes for GWAS identified SNPs. This polygenic priority score (PoPS) also nominated *AHNAK* as a target gene (PoPS = 1.127) for WHR associated SNPs in UKBB GWAS data set. Expression of *AHANK* in adipose tissue was significantly associated with BMI (b= 1.26, p= 6.96 ×10^−14^, Sperman r= 0.44) in AAGMEx. Similarly, *RAP2A* was identified as target gene for BMI associated SNP rs1467693 (cS2G score=1) in UKBB GWAS dataset, and the expression of *RAP2A* in adipose tissue was significantly associated with BMI in AAGMEx.

## Discussion

Studies exploring the role of genetic factors in exosome biology in humans are limited. A recent study measured levels of surface marker proteins of EVs isolated from plasma of 96 participants and identified significant SNPs associated with certain EVs with specific surface protein markers, and EV surface proteins were associated with adiposity-related traits, including waist circumference^47^. Their study provides evidence that EVs with specific surface proteins have phenotypic and genetic links to obesity. In this study we focused on genes involved in biogenesis, assembly, and secretion of exosomes in human adipose and muscle tissues. Utilizing genotype and gene expression data from well-powered African and European ancestry human cohorts, we showed that: 1) expression levels of many “exosome pathway” genes in adipose and muscle tissue are associated with obesity and gluco-metabolic phenotypes, and 2) expression of a subset of these genes are associated with the genotype of common genetic variants. Additionally, in bioinformatic analysis “exosome pathway” genes were identified as target genes for obesity and related gluco-metabolic disease-associated SNPs. Thus, our study suggests that genetic regulation of the exosome pathway may play a key role in pathogenesis of obesity and related gluco-metabolic diseases.

In our study, transcript levels of exosome pathway genes were associated with the genotype of common genetic variants (MAF ≥ 0.01), suggesting a precise genetic regulation of the biogenesis and secretion of exosomes from both adipose and muscle tissues. However, as compared to muscle, transcript level expression of a significantly larger number of “exosome pathway” genes in adipose was correlated with obesity and related gluco-metabolic phenotypes, prompting us to hypothesize that genetic regulation of exosome biogenesis in adipose will likely play a more important role in modulating levels of these tissue-derived exosome in human. One limitation of our study is that we do not provide direct evidence on the effect of manipulating these genetic regulators on exosome levels in support of our hypothesis. However, our study paves the foundation for future functional studies. Manipulating tissue/ cell-specific expression levels of genetically regulated exosome pathway transcripts identified in our study will be required for experiment validation of our hypothesis and for the mechanistic understanding on the role of these genes in exosome biogenesis and secretion.

Genome-wide association studies identified several non-coding SNPs associated with obesity and related gluco-metabolic traits^48^. Mechanistic understanding on the effect of the majority of these SNPs on deranging molecular and cellular mechanisms are currently unknown^49^. Integrating data from GWAS catalogues and functional annotation-based predicated target gene data, our study suggested hitherto unreported mechanism, that is a subset of obesity and related gluco-cardio-metabolic trait associated SNPs are likely involved in causing obesity and insulin resistance via their role in modulating adipose-derived exosome levels and altered cell-cell communication. In our study top adipose-expressed exosome pathway genes positively correlated with BMI includes *RAP2A*. Interestingly integration GWAS data functional genomics-based target gene prediction also identified *RAP2A* as target gene for BMI associated SNP rs1467693 in UKBB GWAS dataset. The RAP proteins are members of the Ras subfamily of small GTPases with divergent activation kinetics and roles in cellular functions. Exosomes are released into extracellular space upon fusion of MVBs with plasma membranes, and this process needs to overcome energy barrier. Protein involved in membrane fusion includes soluble N-ethylmaleimide-sensitive factor attachment protein receptors (SNAREs), tethering factors, Rabs, and other Ras GTPases^31^. Live cell imaging^50^ and shRNA screening studies in HeLa cells^51^ provide evidence in support the role of small GTPases in various steps of exosome secretion. The precise role of *RAP2A* in exosome machinery in adipose tissue remains to be validated, and previous studies suggested the role of *RAP2A* in hormone-stimulated lipolysis in obesity^52^. However, our data suggests that genetic regulation of *RAP2A* expression may putatively cause obesity by altering exosome mediated process.

Our integrative analysis also suggested that expression of AHNAK nucleoprotein is genetically regulated in adipose tissue, and it is a target gene for several WHR associated SNPs. Recent studies suggested that these WHR-associated SNPs are in *AHNAK* enhancer region and alter its expression by affecting binding of transcription factor ELF1 in preadipocytes^53^. Ahnak, is an exceptionally large protein (700 kDa), highly expressed in adipose tissue and is upregulated in high-fat diet-induced obesity models, and Ahnak KO mice showed strong resistance to diet-induced obesity and hepatic steatosis^54^. Previous studies have reported that Ahnak is involved in obesity and cellular adipogenesis process and control adipogenic differentiation by down-regulating Bmpr1α expression in pre-adipocytes.^55^. Studies in human mammary carcinoma cell lines have shown that *AHNAK* is involved in extracellular vesicle biogenesis and secretion^56^ and involved in cell-cell communication. Thus, alternatively genetic regulation of *AHNAK* may be involved in altered adipose tissue-derived exosome mediated cell-cell communication relevant for obesity.

In summary, we show that expression of exosomal genes, especially genes involved in exosome biogenesis, assembly and secretion from adipose and muscle tissue are associated with obesity and insulin resistance. Expression of a subset of these genes are determined by genetic regulatory polymorphisms (eSNPs). Furthermore, analysis of the target genes of GWAS identified gluco-metabolic trait-associated genetic variants suggest that a subset of these SNPs is involved in causing obesity and related gluco-metabolic diseases by modulating the expression of exosome biogenesis pathway genes.

## Supplementary Material

Supplementary Files

This is a list of supplementary files associated with this preprint. Click to download.

- S1SupplementarytablesExosomegenetics522025.xlsx
- S3SupplementarytablesExosomegenetics522025.xlsx
- S4SupplementarytablesExosomegenetics522025.xlsx
- S6SupplementarytablesExosomegenetics522025.xlsx
- S9SupplementarytablesExosomegenetics522025.xlsx
- S5SupplementarytablesExosomegenetics522025.xlsx
- S7SupplementarytablesExosomegenetics522025.xlsx
- SupplementarymaterialexosomegeneticsDasSK2025.docx
- S2SupplementarytablesExosomegenetics522025.xlsx
- S8SupplementarytablesExosomegenetics522025.xlsx

## Figures and Tables

**Table-1:** Exosome pathway genes are predicted targets for obesity GWAS identified SNPs. SNPs are associated with obesity traits in different genome-wide association (GWAS) datasets curated in GWAS catalogue. Target genes predicted by combined SNP-to-gene-linking (cS2G) strategy by Gazal et. al. (2022). Association of the transcript levels of these genes in adipose tissue with BMI and other gluco-metabolic traits based on Sperman’s semi-partial correlation analysis in AAGMEx cohort are shown.

TRAIT	SNP	cS2G Score	Target Gene	Entrez Gene_ID	BMI		Matsuda index		S_I_	
					r	P	r	P	r	P
BMI	rs2076603	0.755	*ATP13A2*	23400	0.08	0.202	−0.19	0.002	−0.09	0.160
BMI	rs3130048	1	*BAG6*	7917	−0.24	9.12E-05	0.20	0.002	0.12	0.072
BMI	rs111612372	1	*SDC1*	6382	0.18	0.005	−0.22	0.001	−0.17	0.012
BMI	rs7640424	1	*CD47*	961	0.16	0.010	−0.08	0.201	0.06	0.375
BMI	rs1561029	1	*CD47*	961	0.16	0.010	−0.08	0.201	0.06	0.375
WHR	rs2509967	0.802	*AHNAK*	79026	0.44	2.00E-13	−0.32	3.60E-07	−0.33	2.84E-07
WHR	rs2428549	0.802	*AHNAK*	79026	0.44	2.00E-13	−0.32	3.60E-07	−0.33	2.84E-07
WHR	rs7200336	0.931	*VASN*	114990	0.20	0.001	−0.17	0.007	−0.04	0.528
WHR	rs2509963	1	*AHNAK*	79026	0.44	2.00E-13	−0.32	3.60E-07	−0.33	2.84E-07
WHR	rs429358	1	*APOE*	348	−0.31	7.27E-07	0.19	0.003	0.26	5.40E-05
WHR	rs12112380	1	*AQP1*	358	0.27	1.29E-05	−0.17	0.007	−0.23	3.52E-04
WHR	rs3747577	1	*VASN*	114990	0.20	0.001	−0.17	0.007	−0.04	0.528
WHR	rs4705986	1	*HSPA4*	3308	0.10	0.103	−0.22	4.56E-04	−0.12	0.068
WHR	rs72801474	1	*HSPA4*	3308	0.10	0.103	−0.22	4.56E-04	−0.12	0.068
WHR adj BMI	rs3738814	0.727	*ATP13A2*	23400	0.08	0.202	−0.19	0.002	−0.09	0.160
WHR adj BMI	rs115942480	1	*TKT*	7086	−0.28	6.08E-06	0.11	0.074	0.07	0.288
WHR adj BMI	rs1330	1	*NUCB2*	4925	0.20	0.002	−0.32	2.74E-07	−0.24	1.86E-04
WHR adj BMI	rs56274609	1	*ATP13A2*	23400	0.08	0.202	−0.19	0.002	−0.09	0.160
WHR adj BMI	rs852425	0.744	*ACTB*	60	0.31	6.01E-07	−0.38	9.85E-10	−0.22	0.001
WHR adj BMI	rs77701059	0.981	*AHNAK*	79026	0.44	2.00E-13	−0.32	3.60E-07	−0.33	2.84E-07
WHR adj BMI	rs757081	1	*NUCB2*	4925	0.20	0.002	−0.32	2.74E-07	−0.24	1.86E-04
WHR adj BMI	rs4786485	1	*VASN*	114990	0.20	0.001	−0.17	0.007	−0.04	0.528
WHR adj BMI	rs3749748	1	*SLC12A2*	6558	−0.13	0.043	0.12	0.067	0.15	0.025
WHR adj BMI	rs3810818	1	*VASN*	114990	0.20	0.001	−0.17	0.007	−0.04	0.528

## Data Availability

The datasets generated during and/or analyzed during the current study are available from the corresponding author on reasonable request. Other information on data availability in public databases is included in the methods section.
